# Unexpected tachycardia and hypertension during anesthetic induction with remimazolam in cardiac surgery: a case report

**DOI:** 10.1186/s40981-021-00462-8

**Published:** 2021-07-28

**Authors:** Tomoe Sato, Sho Ohno, Makishi Maeda, Yasuaki Sawashita, Naoyuki Hirata, Michiaki Yamakage

**Affiliations:** Department of Anesthesiology, Sapporo Medical University School of Medicine, South 1, West 16, Chuo-ku, Sapporo, Hokkaido 060-8543 Japan

**Keywords:** Remimazolam, Cardiac surgery, Adrenergic response

To the Editor

Remimazolam, which is a short-acting benzodiazepine, has been available for general anesthesia in Japan [1, 2]. Previous clinical trials showed that the incident rate of intraoperative hypotension during general anesthesia with remimazolam was lower than that with propofol, while the efficacy of general anesthesia with remimazolam and that with propofol in non-cardiac surgery were comparable [1]. Thus, the effects of remimazolam on hemodynamics may be different from those of existing anesthetics. In this case report, we describe a case in which unexpected tachycardia and hypertension occurred during anesthetic induction using remimazolam in cardiac anesthesia.

## Case presentation

A 53-year-old man (161 cm, 83.6 kg) who had severe aortic valve stenosis and regurgitation due to a bicuspid aortic valve was scheduled for aortic valve replacement and ascending aortic replacement. He had no history and was not taking any medication.

**Fig. 1:**
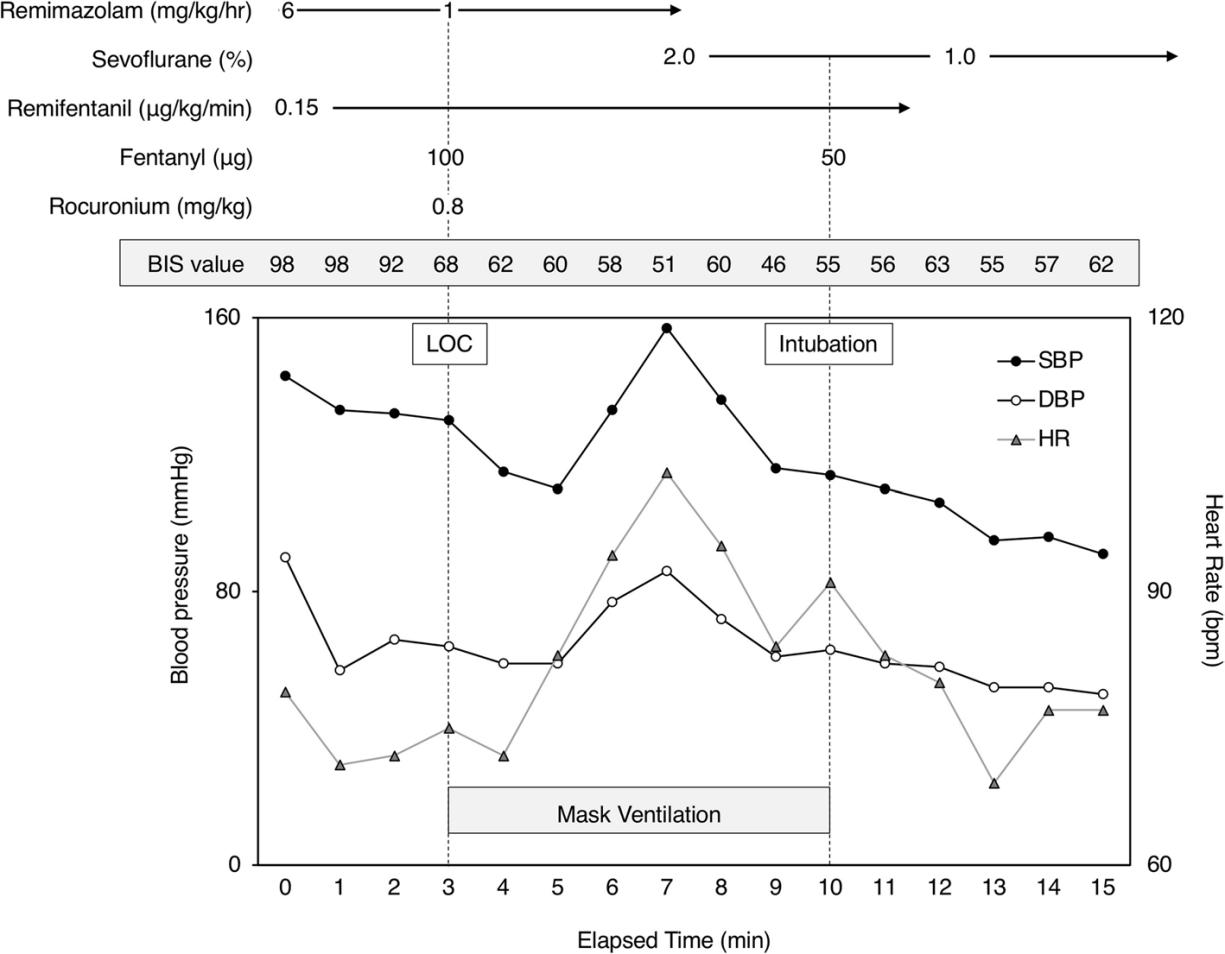
Anesthetic chart during anesthetic induction. LOC, loss of consciousness; BIS, bispectral index; SBP, systolic blood pressure; DBP, diastolic blood pressure; HR, heart rate

Baseline blood pressure (BP) and heart rate (HR) were 143/90 mmHg and 74 bpm with sinus rhythm, respectively. General anesthesia was induced by 6 mg/kg/h remimazolam, 100 μg fentanyl, and 0.15 μg/kg/min remifentanil. After loss of consciousness, the dose of remimazolam was decreased to 1 mg/kg/h and rocuronium (0.8 mg/kg) was administered. Although manual mask ventilation was performed without difficulty and BIS value was 46 to 62 during ventilation, HR gradually increased from 72 to 103 bpm, and BP also increased from 115/59 mmHg to 157/86 mmHg (Fig. 1). At that time, end-tidal carbon dioxide concentration was 35–40 mmHg, and there was no problem with the intravenous line for administration of remimazolam (e.g., occlusion, formation of a precipitation, extravasation). We discontinued remimazolam and started administration of sevoflurane, suspecting that remimazolam was responsible for adrenergic responses. After the end-tidal sevoflurane concentration reached 1.5%, HR and BP decreased to 84 bpm and 115/61 mmHg, respectively, and the BIS value was 46. The patient was intubated about 7 min after administration of rocuronium. After the anesthetic agent had been changed from remimazolam to sevoflurane during anesthetic induction and propofol was administered during cardiopulmonary bypass, there were no unexpected hemodynamic changes throughout the anesthetic period. Surgical procedures were completed successfully. There was no peri-anesthetic awareness and/or recall that could have induced adrenergic responses.

## Discussion and conclusions

We experienced unexpected tachycardia and hypertension during anesthetic induction using remimazolam in a cardiac surgical patient, although remimazolam was used according to the instructions in the package insert.

We believe that intraoperative awareness did not occur in this case because BIS values were appropriate during the occurrence of adrenergic responses. However, the validity of BIS in anesthesia with benzodiazepines has been controversial [3, 4]. Therefore, intraoperative awareness without recall or agitation via inadequate anesthetic depth might have induced the adrenergic responses. Other factors including hypercapnia, stress of manual mask ventilation, and/or the effects of remimazolam on the autonomic nervous system might also have contributed to the adrenergic responses in this case.

Further accumulation of clinical studies and reports on remimazolam would be desirable to increase the clinical reliability of remimazolam for general anesthesia.

## Data Availability

The data that support the findings of this study are available from the corresponding author, N.H., upon reasonable request.

## Abbreviations

BP: Blood pressure
HR: Heart rate
BIS: Bispectral index
